# Book Reviews

**Published:** 1915-04

**Authors:** 


### BOOK REVIEWS

---

Books mentioned may be inspected at and ordered through this office. So far as possible, books received in any month will be reviewed in the issue of the second month following.

---

**The Difficulties and Emergencies of Obstetric Practice,** Comyns Berkeley, M. A., M. D., M. C., F. R. C. P., M. R. C. S. and Victor Bonney, M. S., M. D., B. Sc., F. R. C. S., M. R. C. P. each of London; published by P. Blakiston's Son & Co., Philadelphia and London; second edition, 807 pages, 302 illustrations. $7.50.

In August, 1913, we reviewed the first edition of this work, calling attention not only to its general excellence but to the fact that it economizes time and space by taking for granted the rudimentary principles with which the physician should be familiar. The exhaustion of the first edition indicates the appreciation which it has received from the medical profession. The present edition has been revised and, to some degree, enlarged both in number of pages and of illustrations.

---

**Diseases of the Skin,** James H. Sequeira, M. D., F. R. C. P., F. R. C. S., of London; published by P. Blakiston's Son & Co., Philadelphia; second edition, 650 pages, 48 colored plates, 238 text figures. $8.

After a rather disappointingly brief consideration of the histology and function of the normal skin, the author describes the morphology of elementary lesions and forms of eruptions and proceeds at once to the systematic consideration of dermal pathology, departing from theoretic attempts at analysis for eczema, syphilis and for various groups of diseases in which such attempts would anticipate actual knowledge. We wish that perspiration had been discussed more at length: for instance, the clinician occasionally encounters chromidrosis not due to color-producing bacteria nor to extrinsic dyes but apparently analogous to brick-dust urine and due to the same dietetic and metabolic causes. The mention of the two omissions is, however, really a compliment to the author, for one feels from his scientific and practical discussion of skin diseases in the more literal sense that he would be well qualified to throw side lights on the physiologic and pathologic problems which the skin presents to the internist. The individual discussions are clear and concise, pathology, etiology, etc., being distinguished by black-letter headings of paragraphs. The illustrations are of high order.

**Cancer, Its Cause and Treatment,** L. Duncan Bulkley, A. M., M. D., New York; published by Paul B. Hoeber, 67-69 E. 59, N. Y. 230 pages, $1.50.

The author excludes parasitic cause, contagiousness, heredity, preexisting disease, such as syphilis and malaria, traumatism, telluric causes, although acknowledging the influence of age and admitting the possibility of some relation to trauma. Attention is called to the markedly less susceptibility of animals fed on vegetable proteins, as compared with those eating meat. Schmidt is quoted as having found among 241 cases of cancer of the chylopoietic system, that 180 had never had any infectious disease of childhood and 99 no infectious disease throughout life—indicating that the cancer diathesis, whatever it may be, involves some degree of immunity to ordinary infections. In a discussion racial and geographic distribution and the marked statistic increase of cancer the author leans strongly toward the causation by increase of luxuries and especially in meat consumption. So far as metabolism is concerned, there seems to be some connexion with gouty and lithaemic states and with acidosis in general, which is significant in comparison with the editor's study of statistics which show a marked predilection of cancer for sites having an acid secretion or liable to an acid reaction from fermentation. In his conclusions, the author speaks rather discouragingly of radiation methods, though conceding that they may cause to disappear, a growth due to causes not affected by the emanations. While advocating surgical extirpation, he admits failure in a general average of 90%. Dietetic and other hygienic measures, tending toward a simple life, are advocated as general prophylactics.

---

**Millard Fillmore,** Constructive Statesman. Defender of the Constitution, President of the U. S., by Wm. Elliot Griffis, D. D., L. H. D., of Ithaca. Printed by Andrus & Church, Ithaca.

The author was corporal of the flag-guard of the 44th Po. Volunteers in the Civil War and was a pioneer educator in Japan, 1870-74. All presidents who have succeeded to the presidency by the death of their predecessor are technically "accidents." This fact, the relative lack of opportunity for active political duties by the vice-president, and political differences which are just beginning to have proper perspective, have led to a rather derogatory estimate of Fillmore, even by his fellow-townsmen in Buffalo. This book tends to place him in his proper position as a man of great ability, whose service to the country is no less deserving of appreciation because of the regretable fact that gave him the opportunity to serve it as chief executive. It also emphasizes the tremendous power of the presidency, even in times of peace and without the frank invasion of the legislative field which is now sometimes censured and sometimes regarded as for the best.

---

**Transactions of the Twentieth Annual Meeting of the American Laryngologic, Rhinologic and Otologic Society,** at Atlantic City, June 19-20, 1914. Compiled by Dr. Thomas J. Harris, Secretary, printed by Paul B. Hoeber, N. Y. 362 pages, illustrated.

This contains the papers presented, lists of officers, fellows, etc.; also reports of the section meetings during the past year. The papers are of high order.

---

**Progressive Medicine,** Edited by H. A. Hare and L. F. Appleman, Philadelphia; published by Lee & Febiger, quarterly, $6.00 per annum.

Vol. 17, March, 1915, No. 1. This issue, 382 pages, illustrated, includes surgery of the head and neck, thorax and diseases of the breast, infectious diseases including acute rheumatism, croupous pneumonia and influenza, diseases of children, rhinology, laryngology and otology. It maintains the standard of excellence established, aiming rather to condense into systematic reviews, the principal advances of medical science, than to act as a complete index medicus.

---

**Mortality Statistics,** 1913, published by the Bureau of the Census.

Without attempting a review, we wish to express our acknowledgement of the receipt of this volume and to say that it is available for reference by any of our subscribers.

---

**Clinical Diagnosis.** A Manual of Laboratory Methods. By James Campbell Todd, M. D., Professor of Pathology, University of Colorado. Third edition, revised and enlarged. 12 mo of 585 pages with 176 text-illustrations and 13 colored plates. Philadelphia and London: W. B. Saunders Company, 1914. Cloth $2.50 net.

The blood plate (frontispiece) is excellent though those unfamiliar with blood work should bear in mind that the exact tints vary for different stains, according to degree of washing and unexplainably and that the pictures found clinically are rarely as distinct as in plates. We confess a certain degree of confusion as to large lymphocytes and "phonuclear neutrophilic leucocyte" (uninuclear?) both in practice and in theory and also as to the distinction, in individual cells, between large lymphocytes or uninuclear neutrophiles and myelocytes. This is because many samples of Wright's stain do not give bright red tints where they theoretically should, and because of practical failure to develop typic appearances. The beginner should remember that eosinophiles are distinguished rather by the size and sparkle of the granules than by their actual color; otherwise, he will consider as eosinophiles many ordinary polymorphous cells. He should also be cautious as to diagnosing the "basket type" of disintegrating polymorphous cells as the appearance may be closely simulated by stain distributed over fibrin and debris. The chart of colors in gastric titration opposite page 334 seems to us to need revision. We do not remember ever to have seen phenolphthalein impart the lilac tint to filtrate which is represented. This tint is, indeed, nearer the end reaction with alizarin than the one represented—not to mention the question as to whether the alizarin reaction is at all dependable. Neither does alizarin, previous to neutralization, impart the yellow tint represented. Moreover, the end reaction shown with dimethyl-amido-azo-benzol, occurs about 10 degrees beyond the point at which free HCl should be read. However, it is easy to criticize and difficult to prepare color plates that can be used without actual experience in applied chemistry, microscopic or macroscopic. Taken as a whole, the book is an excellent guide to clinical diagnosis, covering the various topics very completely and with sufficient detail for all but the specialist in laboratory methods. One point deserves emphasis; the small price of the book puts it within reach of all and even for the practitioner who has no time for laboratory work beyond the rudiments, it is worth while to have such a book in the laboratory, both as a guide in cases in which a slight advance beyond the routine is indicated and because the explanations of the different subjects included under the general term of clinical diagnosis, put him in touch with the possibilities of laboratory investigation and show him how to co-operate with the laboratory worker and how much he can expect from such co-operation. The laboratory worker is often embarrassed by expectations far beyond what can be realized and by the receipt of specimens so crudely prepared as to be unavailable. For instance, we have been asked to make a blood count, differentiate types of cells, amount of haemoglobin, from a bit of dried blood clot in a vial.

**Nervous and Mental Diseases.** By Archibald Church, M. D., Professor of Nervous and Mental Diseases in Northwestern University Medical School, Chicago; and Frederick Peterson, M. D. formerly Professor of Psychiatry, Columbia University. Eighth edition, revised. Octavo volume of 940 pages with 350 illustrations. Philadelphia and London: W. B. Saunders Company, 1914. Cloth, $5.00 net; half Morocco, $6.50 net.

The former of the two authors has prepared the part on nervous diseases, the latter, a former Buffalonian, the part on insanity. Save for allotment of chapters whose classification might be doubtful and for close co-operation to avoid conflict and duplication of material and to insure unity of treatment and completeness, each is personally responsible for his own section. In the fifteen years that have elapsed since the first edition, opportunities have been presented for perfection of the work and the inclusion of new material due to a host of students in a rapidly advancing specialty. The mere number of the present edition—the eighth—attests its excellence and the professional appreciation accorded to it and also indicates the repeated opportunities for careful scrutiny of details and improvement of methods of presentation. It may fairly be considered as the standard major treatise of the subject, unsurpassed in English or any other language.

